# Effects of Neuromuscular Electrical Stimulation (NMES) Plus Upper Cervical Spine Mobilization on Forward Head Posture and Swallowing Function in Stroke Patients with Dysphagia

**DOI:** 10.3390/brainsci10080478

**Published:** 2020-07-24

**Authors:** Yung Hyun Jeon, Kyun Hee Cho, Shin Jun Park

**Affiliations:** 1 Department of Physical Therapy, College of Public Health & Welfare, Yongin University, 134, Yongindaehak-ro, Cheoin-gu, Yongin-si, Gyeonggi-do 17092, Korea; 201951313@yiu.ac.kr; 2 AVENS Hospital, 307, Gwanpyeong-ro, Dongan-gu, Anyang-si, Gyeonggi-do 13936, Korea; 201876402@yiu.ac.kr; 3 Department of Physical Therapy, Gangdong University, 278, Daehak-gil, Gamgok-myeon, Eumseong-gun, Chungcheongbuk-do 27600, Korea

**Keywords:** joint mobilization, swallowing dysfunction, NMES, CCFT, dysphagia

## Abstract

After a stroke, forward head posture occurs, resulting in swallowing dysfunction. Neuromuscular electrical stimulation (NMES) combined with upper cervical spine mobilization has demonstrated enhanced recovery of the swallowing function in stroke patients. This study investigated the therapeutic effects of NMES in conjunction with upper cervical mobilization in stroke patients with dysphagia. Thirty-four stroke patients were recruited (in a randomized controlled clinical trial) and divided into an experimental group (*n* = 17; NMES plus upper cervical spine mobilization) and a control group (*n* = 17; NMES plus sham mobilization). Forward head posture was measured by craniocervical flexion test (CCFT) and craniovertebral angle (CVA). Swallowing function was measured by variations in video fluoroscopic dysphagia scale (VDS) and penetration–aspiration scale (PAS) scores using the video fluoroscopic swallowing study (VFSS). All measurements were done at baseline and after four weeks of NMES plus mobilization. A significant increase was observed in CCFT, CVA, VDS (total VDS score, oral stage score, pharyngeal stage score), and PAS score in all variations in the experimental group. The CCFT, CVA, pharyngeal stage score, total VDS, and PAS score were significantly higher in the experimental group when compared to the control group. NMES plus upper cervical spine mobilization can be regarded as a promising method to improve swallowing function and forward head posture changes in stroke patients with dysphagia.

## 1. Introduction

Several stroke patients experience dysphagia during oropharyngeal and esophageal phases [1]. Dysphagia can result in complications, such as aspiration pneumonia, dehydration, malnutrition, and weight loss [2,3]. Thus, therapeutic interventions for improving swallowing in stroke patients is essential [4]. However, despite frequent incidences of dysphagia, therapeutic swallowing interventions are still limited, and there is insufficient evidence that can be used to prevent complications [5].

Swallowing dysfunction is thought to be caused by incorrect postural change [6]. Postural changes that cause swallowing dysfunction include hyper-retraction of the neck [7], excessive kyphosis or lordosis [6], and forward head posture [8]. Particularly, stroke patients may experience aspiration and continued swallowing dysfunction, caused by excessive cervical kyphosis [9]. After discharge from the hospital, 40% of stroke patients use wheelchairs [10]. In fact, thoracic kyphosis and forward head postural changes are observed mainly in the sitting position in stroke patients [11]. Thus, such incorrect postural changes alter swallowing kinetics, which may increase the risk of aspiration [8]. Cervical joint mobilization applied in Alzheimer’s and dementia patients with forward head posture improved the swallowing function [12].

Joint mobilization of the cervical and thoracic spine has been performed in several studies as a desired intervention method for proper alignment of the forward head posture [13,14,15]. Joint mobilization of the spine is referred to as passive movements of joints to achieve the correct position with proper alignment, in order to increase the range of motion or decrease pain [16].

Additionally, joint mobilization evokes reflexogenic effects through stimulation of the articular receptors [17,18,19]. This has been shown to improve the muscle strength around the applied area when applied to joints [17,18,19,20,21]. Cervical spine manipulation may increase anterolateral neck flexion strength [22], and cervical mobilization facilitates cervical muscle contractions and improves swallowing function through proper alignment [12].

Currently, therapeutic swallowing interventions for stroke patients include general dysphagia therapy programs, non-oral feeding, medications, physical and olfactory stimulation [5], electrical stimulation, repetitive transcranial magnetic stimulation (rTMS) [23], transcranial direct current stimulation (tDCS) [24], and neuromuscular electrical stimulation therapy (NMES) [25]. There is a dearth of studies on the effects of incorrect postural changes on swallowing in stroke patients. 

Although joint mobilization is an effective intervention for improving cervical spine posture and swallowing function, the effect of the relationship between swallowing function and cervical spine alignment in stroke patients has not been investigated.

Therefore, the aim of the present study is to investigate the effect of cervical spine mobilization and NMES on cranio-cervical flexion pressure, craniovertebral angle, and swallowing function in stroke patients for a more effective intervention than NMES alone.

## 2. Materials and Methods 

### 2.1. Participants

The study population consisted of stroke patients admitted to a rehabilitation hospital located at Gyeonggi-do. A total of 60 stroke patients (42 male and 18 female) were recruited for the present study on a voluntary basis. Of these, 34 patients satisfied the inclusion criteria. The inclusion criteria were as follows: (1) Korean Mini-Mental State Examination (K-MMSE) score ≥ 19 points, (2) stroke disease duration ≥6 months and <2 years, (3) dysphagia diagnosed by rehabilitation physicians, (4) altered neck posture [11], and (5) voluntary participation. The exclusion criteria were as follows: (1) VitalStim therapy contraindications (pacemakers, dysphagia due to drug toxicity, evidence of nonstop verbalization, significant reflux) [26] and (2) cardiopulmonary disease. 

### 2.2. Sample Size 

Initially, a pilot study was conducted with six subjects. The effect size of cranio-cervical flexion pressure, which was the primary outcome, was 1.11. The total sample size was 28 (14 in the experimental group, 14 in the control group) with an effect size of 1.11, a power of 0.80, and α error probability of 0.05. In total, 34 subjects were selected. G*Power 3.19 (Heinrich Heine University, Dusseldorf, Germany) was used for all sample size calculations [27]. 

### 2.3. Study Procedure

Participants were randomly assigned to two groups. The experimental group (*n* = 17) received upper cervical spine (C1–2) mobilization combined with NMES. The control group (*n* = 17) received upper cervical spine sham mobilization combined with NMES. The experimental and control groups received their respective interventions once a day, three times a week, for 4 weeks. A pre-test was performed before starting the interventions, and a post-test was performed 4 weeks later. Subjects were randomly assigned after the pre-test, and there were no dropouts during the study. Randomization was done by a blinded physical therapist who was independent and not concerned with the treatment and outcome. One of two cards in a box was selected, and patients were assigned to group A (NMES plus upper cervical spine mobilization; experimental group) and group B (NMES plus sham mobilization; control group). The examiner was blinded to the intervention groups of the subjects. The participants were instructed not to discuss any information related to the interventions with other subjects. The research protocol was approved by the Institutional Review Board of Yongin University (2-1040966-AB-N-01-20-2002-HSR-177-2), and all studies were conducted in accordance with the Helsinki Declaration. Informed consent was obtained from all the participants before the study.

### 2.4. Protocol

The study period was 4 weeks. All interventions were performed in a sitting position, and both groups received NMES for 30 min. NMES was performed by three experienced occupational therapists. The experimental and control groups received upper cervical spine mobilization and sham mobilization for 10 min, respectively. Joint mobilization was performed by a physical therapist with more than 160 h of manual therapy education.

#### 2.4.1. NMES

An NMES device was applied to the suprahyoid muscles using VitalStim® (Chattanooga group, Austin, USA). NMES was powered with a dual-channel electrotherapy system with a frequency of 80 Hz, pulse duration of 700 ms, systemic biphasic waves, and intermittent stimulation. Electrodes were attached to the motor point of the suprahyoid muscles (digastric muscle) to induce anterior excursion and vertical elevation movements of the hyoid bone during normal swallowing [28]. Electrodes were placed as follows: two paired electrodes of channel 1 were placed at the left and right sides of the central line between the tip of the chin and hyoid midpoints, and two paired electrodes of channel 2 were placed between the tip of the chin and the angle of mandibles bilaterally [28,29]. The stimulation was applied by gradually increasing the intensity to the level that patients felt a grabbing sensation in the neck without pain or laryngospasm. The therapist confirmed that visible muscle contractions appeared in their necks.

#### 2.4.2. Joint Mobilization

In the forward head posture, upper cervical extension and lower cervical flexion occur simultaneously. Upper cervical spine mobilization improves the flexion movements of C1–C2. The therapist used one hand to hold the subject’s C1 (atlas), placing the thumb and index fingers over the atlas. The other hand was placed on the subject’s occiput (the occiput is held between the hand and shoulder of the therapist) in the direction of suboccipital flexion (based on the convex rule, by moving the occipital condyle posteriorly) [12,16]. The mobilization force could not be standardized. However, since it was applied to all the patients, the therapist performed craniocervical flexion to the point of first resistance and applied slightly more pressure to perform a slight stretch. Cervical mobilization was maintained for 2 min followed by rest for 1 min. A total of three sets were performed in this order.

### 2.5. Assessment

The forward head posture outcomes are the craniocervical flexion test (CCFT) (primary outcome) and craniovertebral angle (CVA). Swallowing function outcomes are the video fluoroscopic dysphagia scale (VDS) and penetration–aspiration scale (PAS). All outcomes were measured during the pre-test and 4 weeks later.

#### 2.5.1. CCFT

Pressure biofeedback (Stabilizer™ Pressure Biofeedback, Chattanooga Group, America) was used to measure CCFT in subjects. Subjects were asked to rest in the supine crook lying position while the neck maintained a neutral position without a pillow. A pressure sensor was placed at the occiput, and 20 mmHg (base pressure) was maintained. CCFT was tested sequentially in five stage pressure levels to test the endurance and deep cervical flexors’ activation in subjects. Five stage pressure levels tested the maintenance of progressive pressure at every 2 mmHg interval from 20 mmHg to a maximum of 30 mmHg. Initially, to determine the pressure levels that could be maintained by the subjects, pressure levels of cranial cervical flexion (the yes movements) where it could be done for a 10-s duration, 10 times were selected. A performance index calculated the number of repetitions that the patients could maintain for 10 s, depending on the pressure level. For example, if a patient was able to maintain craniocervical flexion four times for 10 s at a pressure level of 26 mmHg, the performance index of the patient was calculated as 24 (6 (pressure level) × 4 (number of times)) [30].

#### 2.5.2. CVA

Image J (National Institute of Health, NIH Version, 1.32J, USA) for Windows was used to measure the CVA in patients [31]. The subjects were comfortably seated in a chair, and tragus and C7 spinous processes, serving as reference points, were marked. The subjects were asked to sit in a chair, in a comfortable and natural position, and gaze at a sign in front of them. To minimize parallax errors, the camera was placed vertically with the camera lens facing 80 cm away from the box shoulder. CVA measured the angle where the line connecting the C7 spinous process and tragus and the horizontal line based at C7 intersected [32,33,34].

#### 2.5.3. Swallowing Function Test

The video fluoroscopic swallowing study (VFSS) was used to measure the swallowing function of the subjects. After subjects were seated, 5 mL of barium diluted in water (35% weight/volume) was swallowed, and VFSS was measured. In this test, the video fluoroscopic dysphagia scale (VDS) and penetration–aspiration scale (PAS) were evaluated.

The VDS is divided into oral and pharyngeal stages [35]. The oral stage is composed of seven items with a total score of 40 points: lip closure (Intact, 0; Inadequate, 2; None, 4), bolus formation (Intact, 0; Inadequate, 3; None, 6), mastication (Intact, 0; Inadequate, 4; None, 8), apraxia (None, 0; Mild, 1.5; Moderate, 3; Severe, 4.5), tongue-to-palate contact (Intact, 0; Inadequate, 5; None, 10), premature bolus loss (None, 0; <10%, 1.5; 10%~50%, 3; >50%, 4.5), and oral transit time (≤1.5 s, 0; >1.5 s, 3). The pharyngeal stage is composed of seven items with a total score of 60 points: triggering of pharyngeal wallow (Normal, 0; Delayed, 4.5), vallecular residue (None, 0; <10%, 2; 10%~50%, 4; >50%, 6), laryngeal elevation (Normal, 0; Impaired, 9), pyriform sinus residue (None, 0; <10%, 4.5; 10%~50%, 9; >50%, 13.5), coating of pharyngeal wall (No, 0; Yes, 9), pharyngeal transit time (<1.0 s, 0; >1.0 s, 6), and aspiration (None, 0; Supraglottic penetration, 6; Subglottic aspiration, 12).

PAS is a criterion to evaluate the laryngeal stage based on vocal fold [36]. Assessments are as follows: 1 point (normal), if food does not enter the airway; 2–5 points (penetration), if food is above or at the same level as the vocal fold; or 6–8 points (aspiration), if food is below the vocal fold. In the current study, total VDS score, oral stage score, pharyngeal stage score, and total PAS score were calculated for results.

### 2.6. Statistical Analysis

For statistical analysis of the study, SPSS 20.0 for Windows (IBM Corp., Armonk, NY, USA) was used. For the statistical significance level, α level was set to 0.05. General characteristics of the subjects and a test of homogeneity of pre-test values were analyzed with independent t-test and chi-square tests, and normal distribution was calculated with the K–S test. Results were expressed using the mean and standard deviation. Two-way repeated-measure analysis was performed for the analysis of intervention effects. Within-subject factors (Time) were pre-test and post-test, while between-subject factors (Time * Groups) were the experimental group and control group. When significant differences were observed, an independent t-test (Time * Groups) and paired t-test (Time) were used for post-hoc analysis.

## 3. Results

The general characteristics confirmed that there was no significant difference between the two groups (Table 1).

### 3.1. Change of CCFT and CVA 

A significant between-subject change (F = 4.498, *p* = 0.042) was CCFT. The experimental group showed significantly greater increases in CCFT than in the control group (*P* < 0.05). Significant within-subject change (F = 34.957, *p* = 0.001) was detected. A significant between-subject change (F = 4.408, *p* = 0.044) was CVA. The experimental group showed significantly greater increases in CVA than in the control group (*p* < 0.05). Significant within-subject change (F = 63.328, *p* = 0.001) was detected. The two groups showed significant increases in CCFT and CVA (Table 2).

### 3.2. Change of VDS and PAS 

Significant between-subject changes were VDS total score (F = 4.351, *p* = 0.045) and pharyngeal phase (F = 8.816, *p* = 0.006). The experimental group showed significantly greater increases in VDS total score and pharyngeal phase than in the control group (*p* < 0.05). No significant between-subject change (F = 0.068, *p* = 0.795) was found in the oral phase. Significant within-subject change (F = 55.908, *p* = 0.001) was detected. Significant between-subject change (F = 4.637, *p* = 0.039) was found in PAS. The experimental group showed significantly greater increases in PAS than in the control group (*p* < 0.05). Significant within-subject change (F = 95.459, *p* = 0.001) was detected. The two groups showed significant increases in VDS total score, pharyngeal phase, oral phase, and PAS (Table 3).

## 4. Discussion

In this study, we observed that NMES plus upper cervical spine mobilization, performed to increase the swallowing function in stroke patients, improved the forward head posture and swallowing function. Moreover, this study demonstrated that NMES plus upper cervical spine mobilization is more effective for CCFT and swallowing functions than NMES alone. The findings of this study were consistent with the results of a previous study, which revealed that cervical joint mobilization is effective in improving swallowing function in Alzheimer’s disease patients with dysphasia [12]. 

However, in contrast to the previous study, which did not confirm cervical alignments, the current study has measured changes in CCFT and CVA. Stroke patients have problems with postural control of the trunk, which leads to thoracic kyphosis and forward head posture in the sagittal plane [11]. Forward head posture in stroke patients may lead to swallowing dysfunction and persistent aspiration [9]. Forward head posture is characterized by upper cervical extension and lower cervical flexion. As a result, the length of suprahyoid muscles increase, and the activity of the suprahyoid muscles weaken during swallowing, making it difficult to swallow [37,38]. In this study, upper cervical spine mobilization was applied to increase the craniocervical flexion range of motion. Increasing the craniocervical flexion range of motion leads to increased CCFT, which reflects the contraction of deep cervical flexor muscles [39]. An increase in CCFT due to a cervical alignment change can effectively improve the functioning of swallowing muscles [40]. Therefore, joint mobilization is thought to improve the swallowing function due to C1 segment movements and correct positioning of the cervical spine.

A reclined position with the neck flexed improved the swallowing function in children with cerebral palsy [41]. The results of previous studies that improved the swallowing function in stroke patients after PNF-based short neck flexion exercises support the findings of the current study [42]. 

The upper cervical and upper thoracic spine mobilization applied to subjects with forward head posture was more effective for CVA when compared to deep cervical flexors exercise [13]. 

In studies on joint mobilization to improve forward head posture, cervical and thoracic mobilizations were more effective for CVA and cranial rotation angle (CRA) when compared to isolated cervical mobilization [14]. Upper thoracic joint mobilization increased the cervical curve angle in stroke patients [15]. Joint mobilization in forward head posture was applied to the cervical spine and thoracic spine [13,14]. In the current study, joint mobilization was applied to C1–C2 to improve upper cervical flexion strength. Manipulations done at the same sites as the current study were shown to increase cervical spine muscle strength [22]. Joint mobilization and manipulation increased muscle strength at the site of application through motor unit activity and reflexogenic effects (suppressing inhibitory reflexes) [17,18,19,20,21,22,43]. Therefore, upper cervical joint mobilization can improve the swallowing function by correcting cervical kyphosis and stimulating swallowing muscles in stroke patients.

In this study, CVA was confirmed to change the position of the head against the neck. However, for forward head posture measurement, sagittal head tilt (alternative name: CRA), and sagittal shoulder–C7 angle were used with CVA [44]. Upon consideration, it is very short-sighted to conclude that the forward head posture has been improved only by the increase of CVA. Posture assessments for the head, neck, shoulder, and thoracic regions should be added for a definite conclusion.

Tongue pressure is related to oral dysphagia [45]. NMES in the laryngeal region can increase tongue pressure generated by the contraction of the lingual muscles because it affects hyoid position change [46]. Therefore, NMES can increase the oral phase by increasing the tongue pressure [46] and elevation of the hyoid [29]. This is consistent with the results of previous studies that increased the oral phase after NMES application [47,48]. However, despite our hypothesis that cervical mobilization improves the functioning of the swallowing muscles, it seems to have been difficult to affect tongue pressure. To increase tongue pressure, not only the functioning of swallowing muscles but also hyoid movement and bolus propulsion are required [49]. It seems that there was no difference in the oral phase because cervical mobilization was insufficient to alter the disorder of tongue kinetics.

The strength of this study is that it is the first to attempt cervical spine mobilization for improving the swallowing function in stroke patients. Additionally, this study evaluated cervical flexion strength and the cervical vertebral angle in addition to the swallowing function, which were not measured in previous studies [12]. This indicates the importance of cervical mobilization in swallowing rehabilitation in stroke patients with forward head posture [9]. 

Nevertheless, the present study has several limitations. The current study is very small in sample size. A phase of spontaneous recovery in stroke patients without any intervention cannot be ruled out, so no conclusions can be drawn until a study is conducted with a true control group (without an intervention group) and joint mobilization group alone. Recruitment was limited to mild or moderate stroke patients, and severe forward head posture stroke patients were not included in the study. Therefore, it cannot be generalized to every stroke patient with dysphagia. The posture measurements only included CVA measurement, and change in the activity of each hyoid muscle was not measured. The intervention period was short, lasting only four weeks. Thus, the long-term effects could not be evaluated. Lastly, delicate and skilled hand movements are required to perform joint mobilization. These limitations need to be overcome in future studies. 

## 5. Conclusions

The aim of this study was to evaluate the effect of NMES plus cervical spine mobilization to improve forward head posture and swallowing function in stroke patients. Isolated NMES application improved CCFT and swallowing function only, not CVA. In contrast, the application of NMES plus cervical spine mobilization improved CCFT, swallowing function, and CVA as well, when compared to the isolated NMES application. Therefore, NMES plus upper cervical spine mobilization must be performed to improve CCFT, CVA, and swallowing function in stroke patients with dysphagia. Long-term follow-up studies are required in the future.

## Figures and Tables

**Table 1:** Subject characteristics.

Classification	Experimental Group (*n* = 17)	Control Group (*n* = 17)	*p* ^b^	*p* ^c^
Gender (male/female)	11/6	11/6	0.100	
Paretic side (left/right)	6/11	7/10	0.100	
Hemorrhages/Infarction	14/3	12/5	0.688	
Disease duration (months) ^a^	12.00 ± 2.09	11.82 ± 2.43		0.723
Age (years) ^a^	63.12 ± 13.50	64.47 ± 8.43		0.728
Weight (kg) ^a^	69.11 ± 11.95	65.55 ± 12.66		0.406
Height (cm) ^a^	165.65 ± 9.45	164.58 ± 9.16		0.739
K-MMSE ^d^ (point) ^a^	24.53 ± 2.62	24.24 ± 2.91		0.759
K-NIHSS ^e^ (point) ^a^	10.41 ± 3.06	10.76 ± 3.75		0.766

^a^ Values are denoted as mean ± SD. ^b^ Chi-square test among two intervention groups. ^c^ Independent t-test among two intervention groups. ^d^ K-MMSE: Korean mini-mental state examination. ^e^ K-NIHSS: Korean national institute of health stroke scale.

**Table 2:** Changes in CCFT and CVA outcome of two intervention groups.

Measure/Group	Baseline Test ^a^	Post-Test ^a^	Within-Subject Change ^b^	Between-Subject Change ^b^
CCFT (performance index)				
Experimental group	9.06 ± 4.19	17.53 ± 5.72	8.47 (5.17,11.77) *	4.94(0.81,9.08) **
Control group	8.94 ± 3.94	12.47 ± 4.45	3.53 (0.77,6.29) *	
CVA (degree)				
Experimental group	42.64 ± 4.85	49.49 ± 2.12	6.86 (4.85,8.87) *	2.86(0.36,5.37) **
Control group	42.15 ± 3.98	45.26 ± 3.54	3.11 (1.38,4.84) *	

^a^ Values are means ± SD. ^b^ Values are 95% confidence interval. * Within-subject factors: significant increase from the baseline test. ** Between-subject factors (Interaction): significant increase from the control group. Experimental group: NMES (Neuromuscular electrical stimulation) plus upper cervical spine mobilization. Control group: NMES plus sham mobilization. CCFT: craniocervical flexion test. CVA: craniovertebral angle.

**Table 3:** Changes in swallowing function outcome of two intervention groups.

Measure/Group	Baseline Test ^a^	Post-Test ^a^	Within-Subject Change ^b^	Between-Subject Change ^b^
VDS (point)				
Oral phase				
Experimental group	15.18 ± 4.73	8.97 ± 4.85	−6.21 (−8.43,−3.98) *	−1.85 (−4.73,1.02)
Control group	14.65 ± 5.16	10.29 ± 4.77	−4.35 (−6.35,−2.35) *	
Pharyngeal phase				
Experimental group	43.77 ± 7.24	15.32 ± 7.89	−28.44 (−33.96,−22.93) *	−14.94 (−22.66,−7.23) **
Control group	42.91 ± 7.96	29.41 ± 10.62	−13.50 (−19.33,−7.67) *	
Total score				
Experimental group	58.94 ± 11.22	24.29 ± 7.75	−34.65 (−39.44, −29.86) *	−16.91 (−24.12,−9.71) **
Control group	57.32 ± 11.14	39.71 ± 13.01	−17.62 (−23.45,−11.79) *	
PAS (point)				
Experimental group	4.94 ± 1.64	1.88 ± 0.78	−3.06 (−3.82,−2.30) *	−2.06 (−2.91,−1.21) **
Control group	4.76 ± 1.44	3.76 ± 1.20	−1.00 (−1.45,−0.56) *	

^a^ Values are means ± SD. ^b^ Values are 95% confidence interval. * Within-subject factors: significant increase from the baseline test. ** Between-subject factors (Interaction): significant increase from the control group. Experimental group: NMES plus upper cervical spine mobilization. Control group: NMES plus sham mobilization. VDS: video fluoroscopic dysphagia scale. PAS: penetration–aspiration scale.
